# Effect of Early Postoperative Physical Therapy and Educational Program on Wound Recurrence in Diabetic Foot Ulcers: A Randomized Controlled Trial

**DOI:** 10.3390/jcm14020421

**Published:** 2025-01-10

**Authors:** Shinsuke Imaoka, Genki Kudou, Hikaru Shigefuji, Shion Koujina, Kotaro Matsuki, Taisuke Terou, Shohei Minata

**Affiliations:** Department of Rehabilitation, Oita Oka Hospital, Oita 870-0105, Japan

**Keywords:** diabetic foot ulcer, recurrence prevention, educational program, physical therapy

## Abstract

**Objective:** This study aimed to examine the impact of early postoperative physical therapy combined with an educational program on wound recurrence and quality of life in patients with foot ulcers. **Methods:** Forty-eight postoperative patients with diabetic foot ulcers were randomly assigned to either an intervention group, which received both physical therapy and an educational program (*n* = 25), or a control group, which received physical therapy only (*n* = 23). Each intervention was initiated on the day after surgery in both groups. The intervention group participated in physical therapy sessions, followed by a 15 min disease education program conducted five times per week. The primary endpoint was the rate of wound recurrence six months after hospital discharge. Secondary endpoints included ankle dorsiflexion range of motion, knee extension strength, gait functional independence measure scores, problem areas in diabetes scores, and EuroQol-5 dimensions-5 levels scores. **Results:** The intervention group demonstrated a significantly lower rate of wound recurrence within six months after discharge (10.5%) compared with the control group (27.7%). **Conclusions:** The combined use of early postoperative physical therapy and educational programs was an effective intervention strategy, contributing to reduced wound recurrence rates in patients with diabetic foot ulcers.

## 1. Introduction

A diabetic foot ulcer (DFU) is a condition characterized by infection, ulceration, and/or deep tissue damage in the lower extremity, often associated with neuropathy and impaired peripheral blood flow [1]. In severe cases, 7–20% of DFUs result in lower limb amputation [2], and the 5-year survival rate after amputation is less than 30% [3].

Due to the difficulty in achieving a complete cure and the high recurrence rate, efforts to improve DFU prognosis are increasingly being explored [4,5]. Treatment is further complicated by its multifactorial etiology, which imposes a significant burden on patients, healthcare systems, and society [6]. Even after successful treatment, recurrence is high, with reported rates of 30–40% within the first year [7].

Various patient guidance programs have been developed to prevent DFU recurrence [8,9,10], with some demonstrating a degree of effectiveness. However, these approaches often fall short of achieving consistent preventive efficacy. Limitations in program frequency, methods, and intervention techniques contribute to the lack of consensus regarding optimal strategies [11]. Furthermore, most studies on educational programs focus on outpatient care [12] or home-based foot care [13] for patients with relatively mild wounds, and few reports are available on interventions during hospitalization. 

In cases of severe DFU that require inpatient care, unloading management is a critical component of wound care. However, this approach poses a risk of impaired ability to walk, particularly among elderly patients [14]. Studies have highlighted the effectiveness of physiotherapy during hospitalization in improving walking ability and maintaining quality of life [15]. Despite these benefits, a high rate of re-amputation persists, even when physiotherapy is used to reconstruct gait [16]. Previous studies indicate that even after extended hospitalization and physical therapy aimed at restoring the ability to walk, the risk of short-term recurrence remains substantial. Therefore, we hypothesized that a combination of physical therapy and educational programs aimed at preventing recurrence in the early postoperative period may contribute to the prevention of short-term wound recurrence in patients with severe DFU requiring inpatient care. In this study, we aimed to evaluate the impact of early postoperative physical therapy combined with educational programs on wound recurrence and quality of life in patients with DFU.

## 2. Materials and Methods

### 2.1. Participants

A total of 65 patients required inpatient care for diabetic foot lesions between June 2023 and June 2024. Inclusion criteria included a stable general condition, the ability to participate in continuous physical therapy during hospitalization, cognitive and comprehension skills sufficient to understand simple pamphlets and questionnaires, and independent mobility in a wheelchair at the time of admission. Patients were excluded if they met any of the following criteria: (1) wound infections requiring bed rest, (2) visual impairments preventing the viewing of educational videos, (3) significant hearing impairments that hindered audio comprehension, or (4) a history of major amputation. Each patient received a detailed explanation of the study, both orally and in writing, in accordance with the Declaration of Helsinki. Consent was obtained from all participants. The study received approval from the Ethical Review Board of Oita Oka Hospital (approval no. A-58) and was registered with the National University Hospital Directorate Clinical Trial Registration System (UMIN 000051327). A preliminary power analysis was conducted using G*Power 333 to estimate the required sample size. The main outcome of the study was assumed to be wound recurrence rates, which were compared between the intervention and control groups using the χ^2^ test of independence. Data from a previous study [9] on recurrence prevention in educational and non-educational groups were used as references. To account for a dropout rate of 10%, a minimum of 30 participants per group was determined to be necessary.

### 2.2. Study Protocol

**Figure 1:**
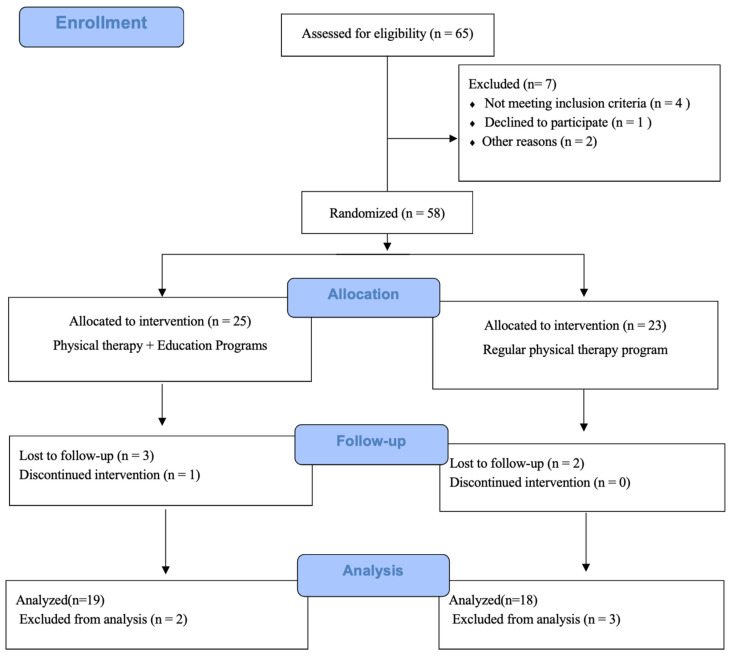
CONSORT (CONsolidated Standards of Reporting Trials) flowchart.

The study utilized a single-blind (rater) block, randomized group comparison trial design. Participants were assigned to either the intervention or control group in a 1:1 ratio using the RAND function (Microsoft Office Excel 2007; Microsoft Corp., Redmond, Washington, DC, USA) (Figure 1). Both participants and their recruiting physicians were blinded to group assignments. Baseline assessments were conducted at admission, and group assignments were completed before initiating the intervention. Due to the nature of the intervention, blinding was not feasible for patients, physicians, physical therapists, or occupational therapists (interventionists and evaluators). All patients underwent regular physical therapy, which included joint mobilization exercises and strength training, performed five times a week beginning the day after surgery and continuing throughout hospitalization. In addition to this standard rehabilitation, the intervention group participated in an educational program aimed at preventing the recurrence of DFU. This program began the day after surgery and was delivered for 14 days by two full-time occupational therapists. The educational content was based on previous studies and focused on key preventive measures: avoiding walking barefoot, using well-fitted shoes, self-monitoring for wounds, seeking referral to specialized medical care in case of trauma, and adhering to foot care regimens. These instructions were supplemented with video content and pamphlets. The sessions were conducted five times per week for 15 min, following the completion of the standard physical therapy sessions.

### 2.3. Outcome Measures

The primary outcome measure was the absence of wound recurrence six months after discharge. Secondary outcome measures included knee extensor strength, ankle range of motion, the problem areas in diabetes (PAID) survey, the EQ-5D-5L (EuroQol-5 dimensions-5 levels), and the functional independence measure (FIM). The rate of wound recurrence, the primary outcome, was evaluated during monthly outpatient follow-ups. Other endpoints were assessed at both the beginning and end of the intervention.

In addition, measurements included participants’ basic information, medical information, and physical function. Basic information included age, sex, body mass index (BMI), requirement (or no requirement) for hemodialysis, time to recurrence, duration of diabetes, need (or no need) for hemodialysis, hospitalization days, amputation region, and comorbidities (lower extremity arterial disease, coronary artery disease).

### 2.4. Primary Endpoint

#### Wound Recurrence

After discharge from the hospital, data were collected from electronic medical records and followed up for six months or until death. Wound recurrence was defined as the reappearance of a wound at the site initially treated during admission. Since our center is the only wound care facility in the prefecture, outpatient follow-up after discharge was exclusively conducted at our institution. Outpatient follow-ups were scheduled at one, three, and six months after discharge.

### 2.5. Secondary Endpoints

#### 2.5.1. Knee Joint Extensor Strength

Maximum voluntary isometric knee extension muscle strength was measured using a hand-held dynamometer (μ-tasF-1; Anima, Tokyo, Japan). Participants were instructed to sit on a chair with their knees flexed at 90° and apply maximum strength against the dynamometer pad for 5 s. Each leg was measured twice, and the highest value recorded for both the right and left legs was used to represent knee extensor muscle strength.

#### 2.5.2. Ankle Joint Range of Motion

The ankle joint was examined in the neutral position with the patient in a supine posture. A vertical line was marked on the patient’s skin from the heel to the midcalf. The maximum range of dorsiflexion in passive motion was measured in degrees with a goniometer.

#### 2.5.3. PAID Survey [17]

The PAID scale is a standardized questionnaire used to assess the emotional and psychological impact of diabetes on individuals. Developed by researchers and healthcare professionals, the scale consists of 20 items that assess various dimensions of emotional distress associated with diabetes management.

#### 2.5.4. EQ-5D-5L [18]

The EQ-5D-5L is a standardized tool developed by the EuroQol Group to measure health-related quality of life. It assesses five dimensions of health: mobility, self-care, usual activities, pain/discomfort, and anxiety/depression. Each dimension is rated across five levels of severity, ranging from no problems to extreme problems, offering a more detailed evaluation compared with earlier versions. The EQ-5D-5L provides a descriptive profile and a single index value for health status. It is widely used in healthcare research, clinical practice, and health economics to assess the effects of health conditions and interventions on patient well-being.

#### 2.5.5. FIM [19]

The FIM is an 18-item, clinician-reported scale used to assess functional ability in six domains, including self-care, continence, mobility, transfers, communication, and cognition. Each item is scored on a scale from 1 to 7, reflecting the level of independence (1 = total assistance required, 7 = complete independence). Ambulation items are rated based on the degree of assistance needed, ranging from 1 point (total assistance: able to walk less than 15 m) to 7 points (full independence: able to walk 50 m without assistance).

### 2.6. Statistical Analysis

All statistical analyses were performed using SPSS version 26.0 (IBM Corp., Armonk, NY, USA). Group differences in age, body mass index, time to recurrence, duration of diabetes, and hospital stay were examined using the independent Student’s *t*-test. The chi-squared test was used to compare sex, recurrence rate, dialysis status, presence or absence of complications, and amputation height. The effect of the intervention on outcome measures was evaluated using a split-plot factorial analysis of variance with a 2 × 2 design (time [preoperative, postoperative] × group [intervention, control]). Bonferroni post hoc tests with two-tailed significance levels were used for specific comparisons.

## 3. Results

### 3.1. Baseline Characteristics

By June 2024, 65 patients were evaluated for eligibility. Of these, 25 were assigned to the intervention group and 23 to the control group (23 patients). The reasons for ineligibility were as follows: (1) five patients had residual postoperative foot infections and were deemed unable to continue physical therapy, (2) four patients had visual impairment caused by retinopathy progression, (3) three patients had cognitive or mental dysfunction, and (4) five patients had a history of major amputation. During the observation period, three patients from the intervention group and two from the control group were excluded due to discharge from the hospital, transfer to another facility, refusal to continue the intervention, or worsening symptoms (Figure 1). The demographic characteristics of both groups are shown in Table 1. No significant differences were found between the intervention and control groups regarding baseline information at admission (age, sex, body mass index, presence of peripheral arterial disease, presence of cardiac disease, dialysis status, duration of hospitalization, diabetes duration, and amputation height).

### 3.2. Within-Group and Between-Group Comparisons

A total of 37 patients were included in the final analysis, with 19 in the intervention group and 18 in the control group. The mean age of the 37 patients was 68.9 years, and 24 (64.8%) were male. Changes in parameters from admission to discharge (within-group comparisons) and between-group differences are shown in Table 2. The dorsiflexion range of motion of the ankle joint was significantly different between the intervention (from 2.8 ± 8.8° to 4.21 ± 8.2°) and control (from 2.7 ± 4.2° to 3.0 ± 6.8°) groups from admission to discharge (*p* = 0.002, η^2^ = 0.2). Similarly, EQ-5D-5L scores demonstrated significant differences between the intervention (from 0.64 ± 0.23 to 0.78 ± 0.16) and control (from 0.74 ± 0.20 to 0.91 ± 0.09) groups from admission to discharge. FIM scores also showed differences between groups (*p* = 0.02, η^2^ = 0.14) and time (*p* < 0.001, η^2^ = 0.53).

By contrast, no significant differences were observed in knee extensor strength between the intervention (from 14.1 ± 5.6 to 15.9 ± 6.5) and control (from 16.9 ± 8.9 to 16.3 ± 6.6) groups from admission to discharge in both time and between groups. PAID scores did not differ significantly between the intervention (from 11.6 ± 11.0 to 19.0 ± 18.9) and control (from 18.3 ± 15.5 to 16.3 ± 15.6) groups or time. No significant interactions were observed for any of the parameters evaluated.

### 3.3. Impact of Combined Physical Therapy and Educational Programs on the Primary Outcome

**Figure 2:**
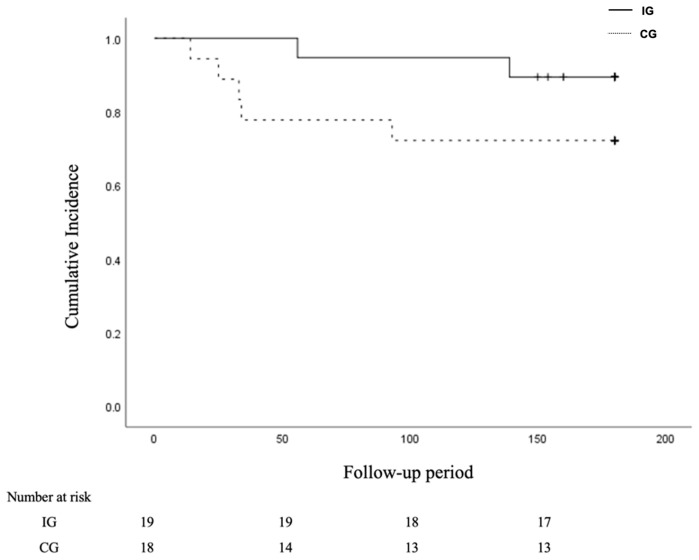
Kaplan–Meier estimates of cumulative recurrence rates of wound. Wound recurrence rates in intervention and control groups of diabetic foot ulcer patients followed for 6 months after discharge from the hospital.

Of the 37 patients included in the final analysis, seven (18.9%) had wound recurrence over a mean follow-up period of 68.4 days. The incidence of wound recurrence was significantly lower in the intervention group, with 2 (10.5%) cases compared with 5 (27.7%) in the control group. The time to wound recurrence was significantly longer in the intervention group, averaging 97.5 (58.7) days compared with 39.3 (30.8) days in the control group (log-rank test, *p* = 0.01; Figure 2).

## 4. Discussion

In this study, the wound recurrence rate during the first six months following hospital discharge was significantly lower in the intervention group, which received both early postoperative physical therapy and an educational program, compared to the control group, which received regular physical therapy. Furthermore, the intervention group exhibited a longer time to wound recurrence (97.5 days vs. 39.3 days in the control group). Previous studies have shown that the six-month wound recurrence rate after hospital discharge is approximately 30% [20,21,22], aligning with the rate observed in the control group. By contrast, the intervention group achieved a wound recurrence rate of 10.5%, which was lower than rates reported in previous studies. These results indicate that a combination of physical therapy and educational programs may reduce wound recurrence and prolong the time to onset. Although some studies described the implementation of educational programs during hospitalization, this is the first to report the impact of an educational program initiated in the early postoperative period. Effective strategies, including continuous monitoring and patient education, are extremely important for preventing DFU recurrence [11]. Regarding patient education and wound recurrence rates, Malone et al. found that educational interventions during hospitalization significantly reduced the incidence of wounds within one year among patients with diabetic foot lesions [23]. A previous study recommended patient education to include instructions on avoiding walking barefoot, using well-fitting shoes, self-monitoring for wounds, seeking referral to a specialized medical facility in case of trauma, and adhering to a foot care regimen. The present study applied these recommendations to the educational intervention’s duration. Corbett et al. found that 10 to 20 min of individualized foot care education significantly improved foot care knowledge and self-care practices [24]. Similarly, Borges et al. conducted a one-month follow-up study comparing an intervention group that received a 15 min educational session with a control group that received no intervention, reporting significant improvements in self-reported behavioral assessment scores [25]. In the present study, a 10 min video-viewing session followed by 5 min of individualized feedback from professional staff was implemented, referring to previous studies. Short, continuous educational guidance could induce behavioral changes in foot care after hospital discharge, consistent with prior research. Regarding the combined effectiveness of patient education and exercise instruction, Pratama et al. found that integrating foot evaluation and educational programs helped prevent recurrence [26]. Another study indicated that combining ankle joint exercises with foot care instruction prevented ulcer recurrence and improved walking ability [27]. In this study, both groups practiced exercise instruction, including resistance exercises for the ankle joint. However, the intervention group showed significantly higher ankle joint dorsiflexion range of motion and gait scores. This result is attributed to the content of the educational video, which included voluntary exercises to maintain ankle range of motion and highlighted the importance of physical activity during walking. The intervention group may have also benefited from conditioning methods practicable in bed, in addition to individualized physical therapy. In addition, previous studies have shown improvements in EQ-5D-5L scores through early rehabilitation [15]. In this study, early rehabilitation was practiced, and the intervention group received instructional content regarding wound management and post-discharge self-care, which may have resulted in significant improvements.

The findings suggest that early postoperative physical therapy combined with patient education may be an effective intervention strategy to prevent wound recurrence within six months after discharge. Previous research has included content regarding the effects of educational guidance after discharge. Based on this, it is anticipated that wound recurrence may be suppressed by providing short, intensive educational guidance during hospitalization and continuing it after discharge.

This study has several limitations. First, the patient population was relatively small, and all participants were Japanese individuals from a single center, which limits the generalizability of the findings. Second, although the study was randomized, included a control group, and employed blinded outcome assessors, it lacked a designated intervention group. Third, the researchers were unable to measure physical activity levels during the postoperative rest period. Therefore, it is possible that the effects of postoperative disuse syndrome influenced recurrence. Fourth, the survey was limited to patients with type 2 diabetes and lacked basic information (educational history and glycemic control information). Despite these limitations, the study demonstrated that early postoperative patient education and instruction may help prevent wound recurrence within six months after hospital discharge. The combination of patient education and exercise therapy in the early postoperative period seems to play an important role in preventing wound recurrence and maintaining physical function. This approach may represent a useful intervention strategy to prevent recurrence.

## 5. Conclusions

Our results suggest that early postoperative education for patients with diabetic foot ulcers may reduce the rate of wound recurrence within six months after hospital discharge. Physical therapy combined with patient education during the postoperative rest period seems to be an effective strategy for preventing wound recurrence. Patient education would also benefit from a smooth introduction in the early postoperative period due to its less invasive nature.

## Figures and Tables

**Table 1:** Baseline characteristics of patients.

Characteristics	Intervention		Control		*p*-Value
	Group		Group		
	(N = 19)		(N = 18)		
Age (years)	70.4	(12.7)	67.9	(9.7)	0.50
Male, n (%)	12	63.1	12	66.6	0.83
BMI (kg/cm^2^)	24.3	(5.1)	23	(3.8)	0.53
Recurrence, n (%)	2	10.5	5	27.7	0.18
Time to recurrence (days)	97.5	(58.7)	39.3	(30.8)	0.03
Duration of diabetes (years)	16.1	(7.5)	18.9	(4.0)	0.47
Hemodialysis, n (%)	10	52.6	8	(44.4)	0.25
Comorbidities					
LEAD, n (%)	8	42.1	10	(55.5)	0.41
CAD, n (%)	4	21.0	3	(16.6)	0.37
Length of stay	40.5	(25.3)	53.3	(33.3)	0.13
Amputation region, n (%)					
Toe amputation	14	73.6	13	72.2	0.43
Ray amputation	4	21.0	3	16.6	
Transmetatarsal amputation	1	5.2	2	11.1	

Data are mean (SD) or median (Q1–Q3) unless otherwise indicated. Independent *t*-test. Chi-squared test. BMI, body mass index; LEAD, lower extremity artery disease; CAD, coronary artery disease.

**Table 2:** Descriptive and inferential statistics of the primary outcomes.

Variable	Assessment	Intervention	Control	Mean	Repeated Measure ANOVA			
	Time	Group	Group	Difference				
		(n = 19)	(n = 18)	95% CI		*F*	*p*	η^2^
Ankle dorsiflexion range (degrees)	Pre	2.89 ± 8.86	2.78 ± 4.27	1.65 (−0.58 to 6.13)	Interaction	0.265	0.61	0.018
	Post	4.21 ± 8.21	3.06 ± 6.89	1.79 (−0.58 to 6.69)	group	0.085	0.002	0.2
					time	0.624	0.435	0.008
Knee joint extensor strength (kg)	Pre	14.1 ± 5.6	16.9 ± 8.9	0.01 (−0.07 to 0.07)	Interaction	1.628	0.21	0.012
	Post	15.9 ± 6.5	16.3 ± 6.6	0.06 (0.06 to 0.10)	group	0.576	0.453	0.016
					time	0.415	0.524	0.012
FIM ambulation (points)	Pre	2.56	1	0.35 (2.79 to 4.21)	Interaction	1.07	0.3	0.03
	Post	4.44	3.63	0.31 (1.62 to 3.00)	group	5.88	0.02	0.14
					time	39.77	<0.001	0.532
EQ-5D-5L (points)	Pre	0.64 ± 0.23	0.74 ± 0.20	0.01 (−0.09 to 0.07)	Interaction	0.2	0.65	0.006
	Post	0.78 ± 0.16	0.91 ± 0.09	0.01 (−0.10 to 0.08)	group	6,14	0.018	0.14
					time	18.7	<0.001	0.34
PAID (points)	Pre	11.6 ± 11.0	18.3 ± 15.5	3.17 (12.3 to 25.2)	Interaction	6.172	0.18	0.15
	Post	19.0 ± 18.9	16.3 ± 15.6	2.89 (11.8 to 23.6)	group	0.22	0.64	0.007
					time	1.57	0.21	0.04

Values are expressed as means ± SD. Analysis was performed using a split-plot factorial analysis of variance, followed by the Bonferroni post hoc test. FIM, functional independence measure; EQ-5D-5L, EuroQol-5 dimensions-5 levels; PAID, problem areas in diabetes survey.

## Data Availability

The datasets generated and analyzed during the current study are available from the corresponding author upon reasonable request.
